# PINPOINT® can be used for photodynamic diagnosis based on 5-aminolevulinic acid-induced protoporphyrinIX in gastric cancer surgery: Report of a case

**DOI:** 10.1016/j.ijscr.2020.03.008

**Published:** 2020-03-09

**Authors:** Teppei Kamada, Masashi Yoshida, Hironori Ohdaira, Sojun Hoshimoto, Satoshi Narihiro, Norihiko Suzuki, Rui Marukuchi, Hideyuki Takeuchi, Yutaka Suzuki

**Affiliations:** Department of Surgery, International University of Health and Welfare Hospital, 537-3, Iguchi, Nasushiobara, Tochigi, 329-2763, Japan

**Keywords:** PDD, photodynamic diagnosis, 5-ALA, 5-aminolevulinic acid, dPTEG, double percutaneous transesophageal gastrotubing, PpIX, protoporphyrin IX, 5-ALA-PDD, PINPOINT, Aladuck LS-DLED, Pyloric stenosis, PTEG

## Abstract

**Introduction:**

Photodynamic diagnosis (PDD) using 5-aminolevulinic acid (5-ALA) has been used as a diagnostic procedure for malignant diseases. Dedicated laparoscopes (e.g., an IMAGE1 Camera System®) are used for this procedure. We report a case treated with laparoscopic total gastrectomy with 5-ALA-PDD using the PINPOINT® system.

**Presentation of case:**

A patient in his 80 s with diffuse-type gastric cancer with pyloric stenosis and ascites was admitted to our hospital. Double percutaneous transesophageal gastrotubing (dPTEG) for both gastric decompression and enteral nutrition and two cycles of preoperative chemotherapy with S-1 plus oxaliplatin were performed preoperatively.

Additionally, we preoperatively performed an ex vivo experiment that confirmed that the PINPOINT® system can be used to observed protoporphyrin IX (PpIX) fluorescence.

Three hours before surgery, 5-ALA hydrochloride was administered through dPTEG. Observation was performed by PINPOINT®, and Aladuck® was used as an excitation light source.

Peritoneal nodules and sampled lymph nodes with red fluorescence were observed by 5-ALA-PDD. Accordingly, we gave up a radical operation and laparoscopic total gastrectomy without systematic lymphadenectomy to improve anemia and release pyloric stenosis was performed. The patient’s postoperative course was uneventful.

**Discussion:**

It is possible that the connection with each of the scopes and an exclusive light source (Aladuck®) enable the easy use of 5-ALA-PDD without dedicated laparoscopy.

It is expected that 5-ALA-PDD would show the further spread of gastrointestinal cancer if it could be performed with many types of laparoscopes.

**Conclusion:**

We found that 5-ALA-PDD-guided surgery can be easily performed in a short time using the PINPOINT® system.

## Highlights

• We have reported the case that underwent laparoscopic total gastrectomy with photodynamic diagnosis based on 5-aminolevulinic acid induced protoporphyrinIX by using PINPOINT®.
• PINPOINT®, a brightfield color fluorescence camera, could be used for 5-ALA-PDD.
• Administration of 5-ALA through PTEG for a patient with pyloric stenosis was successfully performed.

## 1 Introduction

Photodynamic diagnosis (PDD) performed using 5-aminolevulinic acid (5-ALA) has been used as a diagnostic procedure in malignant diseases and requires the use of a fluorescence laparoscopic system capable of providing 405-nm excitation light through a dedicated rigid laparoscope (e.g., an IMAGE1 Camera System®, KARL STORZ, Tuttlingen, Germany) [1]. However, our search of the literature revealed no previously reported cases in which 5-ALA-PDD was performed using a nondedicated laparoscope (e.g., PINPOINT®).

Protoporphyrin IX (PpIX) emits fluorescence with a peak at 635 nm upon excitation. Because a 635 nm wavelength is visible, it might be possible to observe it under a normal laparoscope.

However, individual scopes possess a variety of optical properties, and nobody knows whether a PINPOINT® (Stryker, Kalamazoo, MI, USA) device would be suitable for observing PpIX fluorescence. The intrinsic amino acid 5-ALA is intimately involved in heme synthesis in nucleated cells. Cancer cells exhibit alterations in enzymatic activity in the heme synthetic pathway. Accordingly, an increase in the activity of porphobilinogen deaminase and a decrease in the activity of ferrochelatase causes the intracellular accumulation of PpIX in cancer cells [2,3].

The amino acid 5-ALA-PDD is now widely used in the fields of neurosurgery (malignant glioma) [4] and urology (urothelial carcinoma of the bladder) [5].

In the field of gastrointestinal cancer treatment, 5-ALA-PDD has been used for the in vivo detection of peritoneal dissemination in patients with advanced gastrointestinal cancers [[6], [7], [8]]. In addition, the feasibility of 5-ALA-PDD in detecting lymph node metastases has been validated in clinical trials [[7], [8], [9], [10]]. Thus, the availability of 5-ALA-PDD for these uses is increasingly anticipated since there are no other cancer-specific fluorophores applicable to clinical use [11].

In 2013, PINPOINT® was introduced for ICG fluorescence-guided surgery; however, it can also be used to observe normal white light. Here, we sought to use PINPOINT® to visualize 5-ALA-PDD in the case of a patient in whom 5-ALA-PDD was used with PINPOINT® for advanced gastric cancer. The work has been reported in line with the SCARE criteria [12].

## 2 Presentation of case

### 2.1 Preoperative preparation

Prior to the operation, we performed an ex vivo experiment to determine whether the PINPOINT® system could be used for PpIX fluorescence observation.

1. We confirmed that Aladuck LS-DLED® (SBI Pharmaceuticals, Tokyo, Japan), an excitation light source (Violet LED with a peak at 400–410 nm), could be connected with the PINPOINT® system (Fig. 1a).

**Fig. 1:**
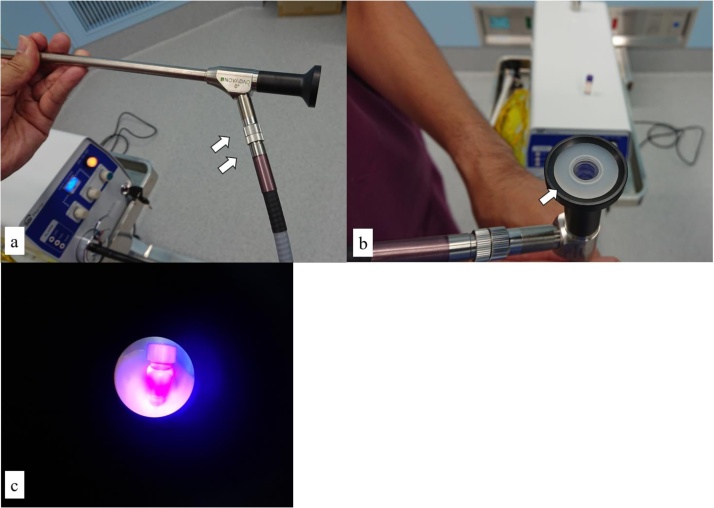
(a) Aladuck LS-DLED®, the light source used for excitation, was connected with the PINPOINT® system. (b) We placed a longpass filter that cut off light with a wavelength of 400-410 into the PINPOINT® system. (c) We observed fluorescence of PpIX using the PINPOINT® system.
2. We placed a filter (SBI Pharmaceuticals, Tokyo, Japan) that cut off wavelengths of 450 nm or less between the rigid laparoscope and the camera head to exclude blue excitation light so that red fluorescence could be observed clearly (Fig. 1b).
3. We set the PINPOINT® system to color HD mode (the mode appropriate for white light full-color images) and turned on the light source of the PINPOINT that was not connected.
4. We observed fluorescence for PpIX (10 μg/ml in solvent: DMSO: dimethyl sulfoxide) using the PINPOINT® system (Fig. 1c).

### 2.2 Case

A man in his 80s with chief complaints of anorexia and weight loss visited our hospital and was diagnosed with diffuse-type advanced gastric cancer with pyloric stenosis and ascites. Peritoneal nodules could not be identified by computed tomographic scanning. He underwent double percutaneous transesophageal gastrotubing (dPTEG) for both gastric decompression and enteral nutrition, as reported by Iwase et al. [13] Oxaliplatin was administered intravenously at a dose of 150 mg/m^2^ on day 1. S-1 was administered enterally at a dose of 100 mg/m^2^ daily from day 1 to 14 of a 3-week cycle. The patient underwent laparoscopic total gastrectomy after a decrease in ascites was confirmed following two cycles of preoperative chemotherapy with S-1 plus oxaliplatin. PDD was performed using 5-ALA as approved by the clinical ethics committee of our Hospital (Approval No.180731).

Three hours before surgery, 5-ALA hydrochloride (SBI Pharmaceuticals, Tokyo, Japan) was dissolved in water at a dose of 20 mg/kg and administered enterally via dPTEG. Observation was performed with a PINPOINT® system in color HD mode, with Aladuck LS-DLED® (SBI Pharmaceuticals, Tokyo, Japan) used as an excitation light source.

**Fig. 2:**
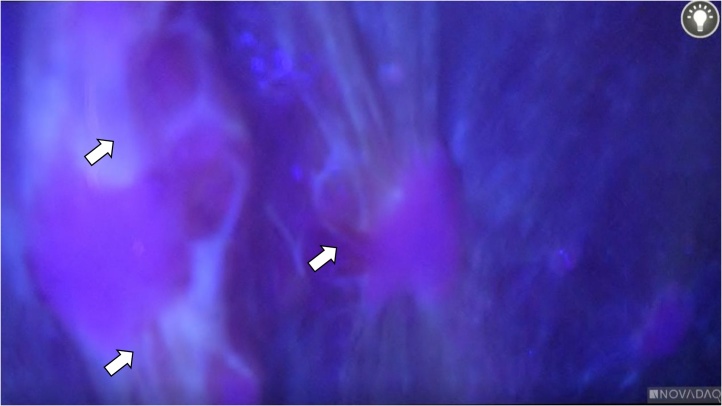
Representative images of 5-aminolevulinic acid-based photodynamic diagnosis. Peritoneal dissemination (arrows): red fluorescence (laparoscopic image).

Peritoneal nodules with red fluorescence were observed using 5-ALA-PDD and diagnosed as peritoneal dissemination (Fig. 2). We gave up a radical operation and total gastrectomy without systematic lymph node adenectomy to improve anemia and release pyloric stenosis was performed. On a back table, the excised specimen was observed intraoperatively.

**Fig. 3:**
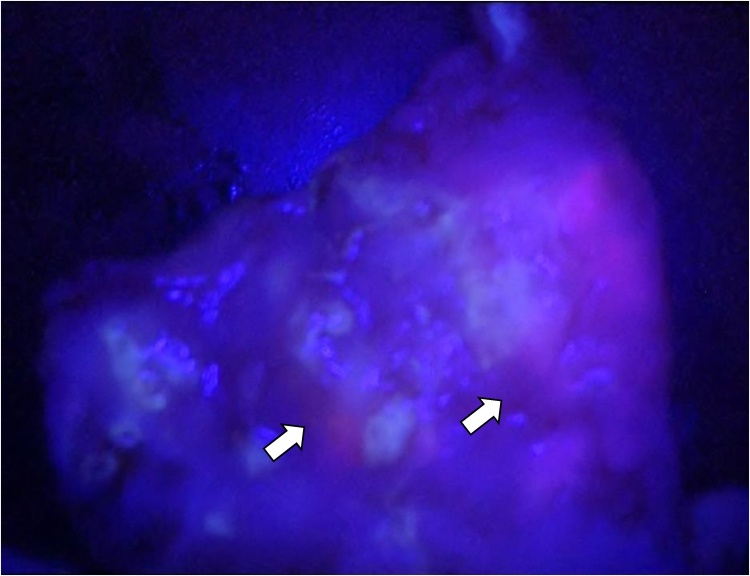
Representative images of 5-aminolevulinic acid-based photodynamic diagnosis. Lymph node metastasis (arrows): red fluorescence (resected specimen).

**Fig. 4:**
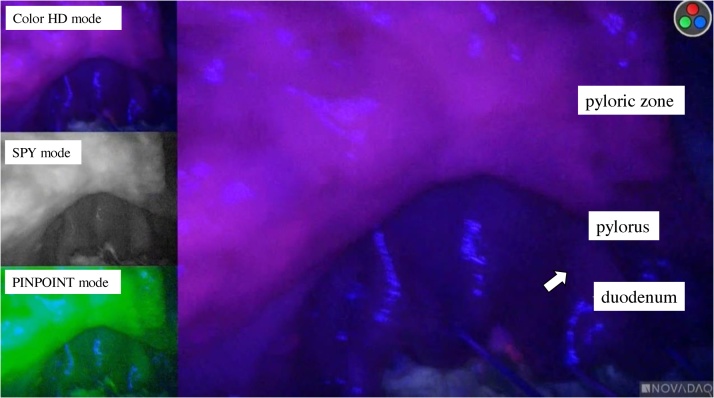
Representative images of 5-aminolevulinic acid-based photodynamic diagnosis. Duodenal invasion of primary gastric cancer (arrows): red fluorescence (resected specimen).

Red fluorescence was observed in a sampled lymph node, and the patient was diagnosed with lymph node metastasis (Fig. 3). The red fluorescence spread slightly from the stomach over the gastroduodenal junction, indicating minor duodenal invasion (Fig. 4).

The pathological diagnosis was moderately differentiated adenocarcinoma, T4a, N3b, M1(P1b), stage IV.

The patient’s postoperative course was good with no complications.

## 3 Discussion

We used PpIX fluorescence diagnostics to observe advanced gastric cancer using a PINPOINT® system connected with Aladuck LS-DLED® as a light source.

Several studies have used 5-ALA-PDD for the diagnosis and treatment of gastric cancer; these included the application of this approach for staging laparoscopy [[7], [8], [9],[14], [15], [16]].

Murayama et al. [8] evaluated the availability of 5-ALA-PDD. Their trial consisted of 13 patients, 5 of whom were diagnosed with peritoneal dissemination by 5-ALA-PDD. These diagnoses were confirmed by subsequent histopathological examination. Whereas 14 peritoneal nodules with suspected dissemination were detected under white light observation, only 12 nodules showed red fluorescence when viewed using 5-ALA-PDD. Histopathological examination of the samples indicated that only the 12 nodules with red fluorescence were disseminated. Therefore, the authors determined that the diagnostic accuracy of 5-ALA-PDD was better than that of white light imaging [8].

Kishi et al. [7] demonstrated the usefulness of adding 5-ALA-PDD to conventional white light laparoscopy in another clinical trial of 52 advanced gastric cancer patients. The authors reported that when white light observation was performed, 24 of the 52 patients showed no macroscopic evidence of peritoneal dissemination, but when 5-ALA-PDD was used, dissemination was detected in 5 of these 24 patients. Thus, the authors concluded that 5-ALA-PDD improved the sensitivity of the detection of peritoneal dissemination [7].

Furthermore, Koizumi et al. [9] conducted a clinical trial of 14 patients with advanced gastric cancer in which they examined a total of 144 lymph nodes.

Metastatic lymph nodes exhibit a distinct red fluorescence consistent with the distribution of a metastatic focus. Although some nonmetastatic lymph nodes were also fluorescent, those nodes could be distinguished from metastasis-bearing lymph nodes by their characteristic follicular fluorescence pattern. When they used a diagnostic algorithm to evaluate fluorescence patterns, Koizumi et al. [9] found that 5-ALA-PDD achieved an acceptable diagnostic power with 92.4 % accuracy.

However, our search of the literature revealed no previously reported cases in which 5-ALA-PDD was used with a PINPOINT® system.

The PINPOINT system allows the simultaneous display of multiple images, including standard high-definition white light images, SPY images (monochromatic images), and pinpoint images (fluorescence images combined with full color images) [17].

Dedicated laparoscopes (e.g., an IMAGE1 Camera System®, KARL STORZ, Tuttlingen, Germany) are used for the amino acid 5-ALA-PDD.

However, in this case, without a dedicated laparoscope, connecting each of the scopes with an exclusive light source (Aladuck LS-DLED®) enabled us to easily use 5-ALA-PDD.

Furthermore, we focused on the fact that 5-ALA-PDD could enable us to shorten the operation time in high-risk or aged patients and present an alternative to intraoperative rapid histopathological diagnosis.

Hence, the clinical ethics committee of International University of Health and Welfare Hospital allowed us to perform 5-ALA-PDD in this patient.

When performing lower invasive surgeries, a rapid and precise method is required to diagnose the presence or absence of lymph node metastases intraoperatively [18].

The existing method for intraoperative rapid histopathological diagnosis is based on frozen sections and has several liabilities. One is the amount of time required to make a histological section. Another is that small metastases might not be detected because only a limited number of histological sections can be made, resulting in a relatively high proportion of false-negatives [19].

In this case, during the operation, duodenal invasion that could not have been diagnosed before the operation and lymph node metastases were identified within a short time because the resected specimens could be observed without the need for rapid intraoperative histopathological diagnosis.

Regarding the future application of 5-ALA-PDD for gastric cancer, it could be used as a tool for evaluating surgical resection margins and could thereby assist with pathological diagnosis during surgery [20].

A limitation of 5-ALA-PDD is reported that gastric cancer patients with accompanying pyloric stenosis should be excluded from procedures because the efficient absorption of 5-ALA is impossible in these patients [11]. However, in this case of gastric cancer with pyloric stenosis, it was possible to use 5-ALA-PDD by administering it enterally via dPTEG. Accordingly, for patients with oral feeding difficulty, administration of the solution enterally via dPTEG might be a useful option.

Additional research will be necessary in the future to determine whether any other scopes, including flexible scopes, could be useful for 5-ALA-PDD. We expect the use of 5-ALA-PDD in the treatment of gastrointestinal cancer to expand if other kinds of laparoscopes could be used with it.

## 4 Conclusion

We report a case in which 5-ALA-PDD was used with the PINPOINT® system. We found it was possible to perform 5-ALA-PDD-guided surgery using the PINPOINT® system easily in a short time.

## Sources of funding

We have no sponsors.

## Ethical approval

This study was approved (Approval No.180731) by the Clinical Ethics Committee of International University of Health and Welfare, Tochigi, Japan.

## Consent

Written informed consent was obtained from the patient for publication of this case report and any accompanying images.

## Author contribution

TK: study design, data collection, data analysis, writing.

MY: critical revision

YS: final approval of the article

Any other authors: data collection

All authors read and approved the final manuscript.

## Registration of research studies

This paper is case report. The authors don’t need to register this work.

## Guarantor

Teppei Kamada, the corresponding author of this manuscript accept full responsibility for the work and the conduct of the study, access to the data and controlled the decision to publish.

## Provenance and peer review

Not commissioned, externally peer-reviewed.

## Declaration of Competing Interest

There are no conflicts of interest.
